# SMC complexes organize the bacterial chromosome by lengthwise compaction

**DOI:** 10.1007/s00294-020-01076-w

**Published:** 2020-04-16

**Authors:** Jarno Mäkelä, David Sherratt

**Affiliations:** Department of Biochemistry, University of Oxford, Oxford, OX1 3QU UK

**Keywords:** Chromosome organization, *Escherichia coli*, SMC complex, MukBEF

## Abstract

Structural maintenance of chromosomes (SMC) complexes are ancient and conserved molecular machines that organize chromosomes in all domains of life. We propose that the principles of chromosome folding needed to accommodate DNA inside a cell in an accessible form will follow similar principles in prokaryotes and eukaryotes. However, the exact contributions of SMC complexes to bacterial chromosome organization have been elusive. Recently, it was shown that the SMC homolog, MukBEF, organizes and individualizes the *Escherichia coli* chromosome by forming a filamentous axial core from which DNA loops emanate, similar to the action of condensin in mitotic chromosome formation. MukBEF action, along with its interaction with the partner protein, MatP, also facilitates chromosome individualization by directing opposite chromosome arms (replichores) to different cell halves. This contrasts with the situation in many other bacteria, where SMC complexes organise chromosomes in a way that the opposite replichores are aligned along the long axis of the cell. We highlight the similarities and differences of SMC complex contributions to chromosome organization in bacteria and eukaryotes, and summarize the current mechanistic understanding of the processes.

## Overview

**Fig. 1:**
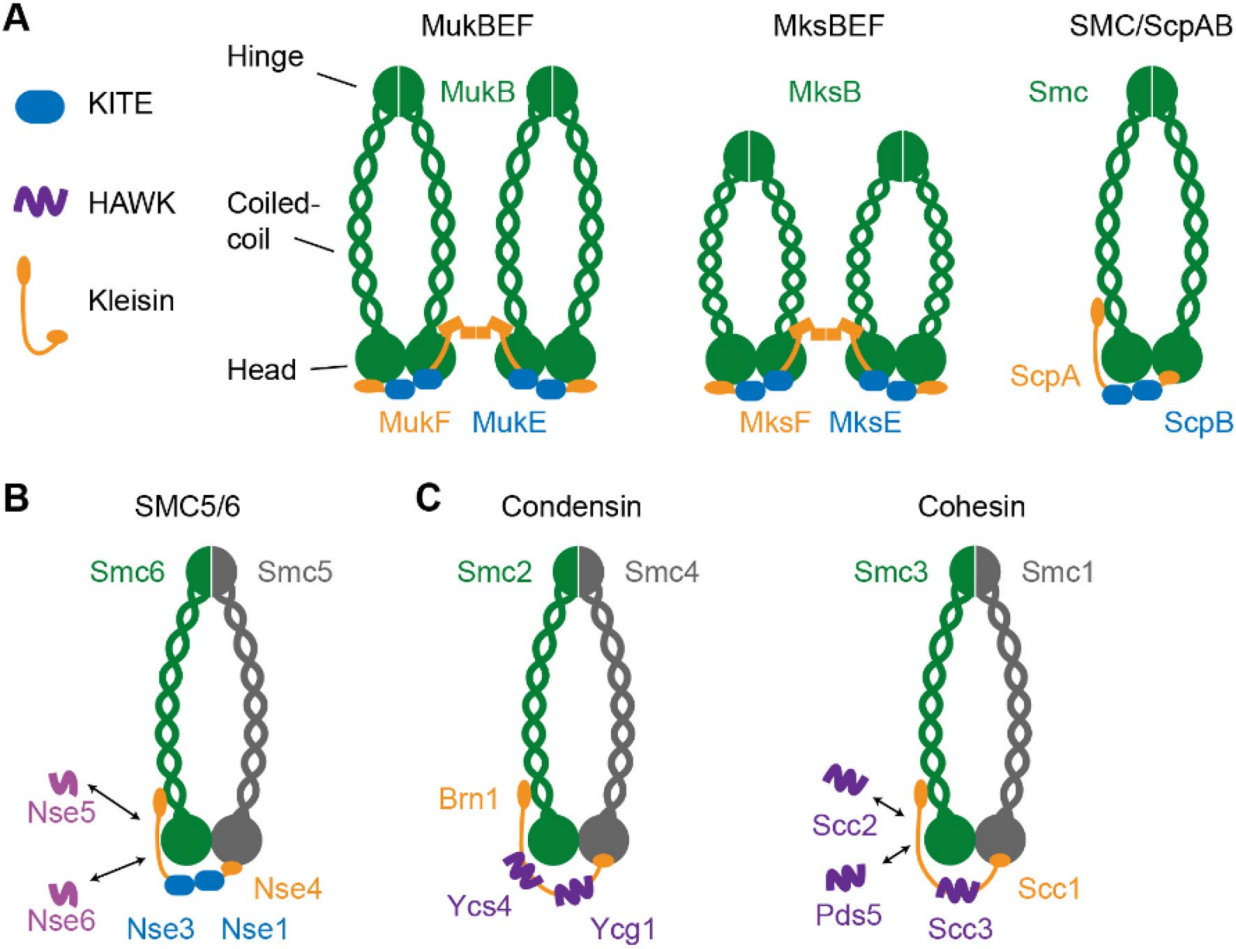
Schematic illustrating SMC architectures*.* SMC proteins contain a head ATPase domain and a hinge dimerization domain, separated by an antiparallel intramolecular coiled-coil. An extended kleisin molecule joins the two ATPase heads of an SMC (hetero) dimer. Either two KITE proteins bind the kleisin [bacterial SMCs (**a**) and eukaryote SMC5/6 (**b**)], or at least two large HAWKs bind cohesin and condensin kleisin (**c**). A non-KITE non-HAWK Nse5/6 heterodimer also interacts with SMC5/6 (Palecek and Gruber 2015). The dimeric kleisin of MukF/MksF leads to dimer of dimer formation, dependent on ATP binding, head engagement and MukBEF. Distant relations to the SMC complexes above are Rad50 and RecN, involved in repair of double-strand DNA breaks (not shown). For further details see (Kakui and Uhlmann 2018; Wani et al. 2018; Paul et al. 2019; Yatskevich et al. 2019)

In all domains of life, incredibly long genomic DNAs must be folded into higher order looped structures to accommodate DNA inside a cell. The processes that manage DNA, whether it be replication, repair, gene expression, or chromosome segregation, must act on DNA in a way that the processes sense whether they are acting on the same or different molecules by tracking the three-dimensional path of individual DNA molecules. A single class of conserved and ancient proteins, structural maintenance of chromosomes (SMC) complexes, play multiple important roles in chromosome organization and individualization [reviewed in (Yatskevich et al. 2019)]. Although *Escherichia coli* MukB was the first SMC protein to be identified through its role in chromosome segregation (Hiraga et al. 1989), much subsequent work has focussed on eukaryote condensins and cohesins, initially implicated in mitotic chromosome compaction and sister chromosome cohesion, respectively. The distinctive architectures and classes of SMC complexes are shown schematically in Fig. 1. Given the conservation of SMC complexes, it is unescapable to propose that they share common mechanisms of action, although these mechanisms have remained elusive and controversial. Nevertheless, recent demonstrations that both purified condensin and cohesin can extrude DNA loops, dependent on ATP hydrolysis, in single-molecule assays in vitro (Ganji et al. 2018; Davidson et al. 2019; Kim et al. 2019, 2020), thereby supporting the earlier proposal that loop extrusion could explain both chromosome organization and individualization (Nasmyth 2001). Remarkably, the importance of chromosome organization and individualization was addressed by Flemming and Boveri, respectively, more than 100 years ago, before the role of chromosomes as the carriers of genetic material was established [see (Yatskevich et al. 2019)].

### Chromosome organization in *E. coli* and other bacteria

**Fig. 2:**
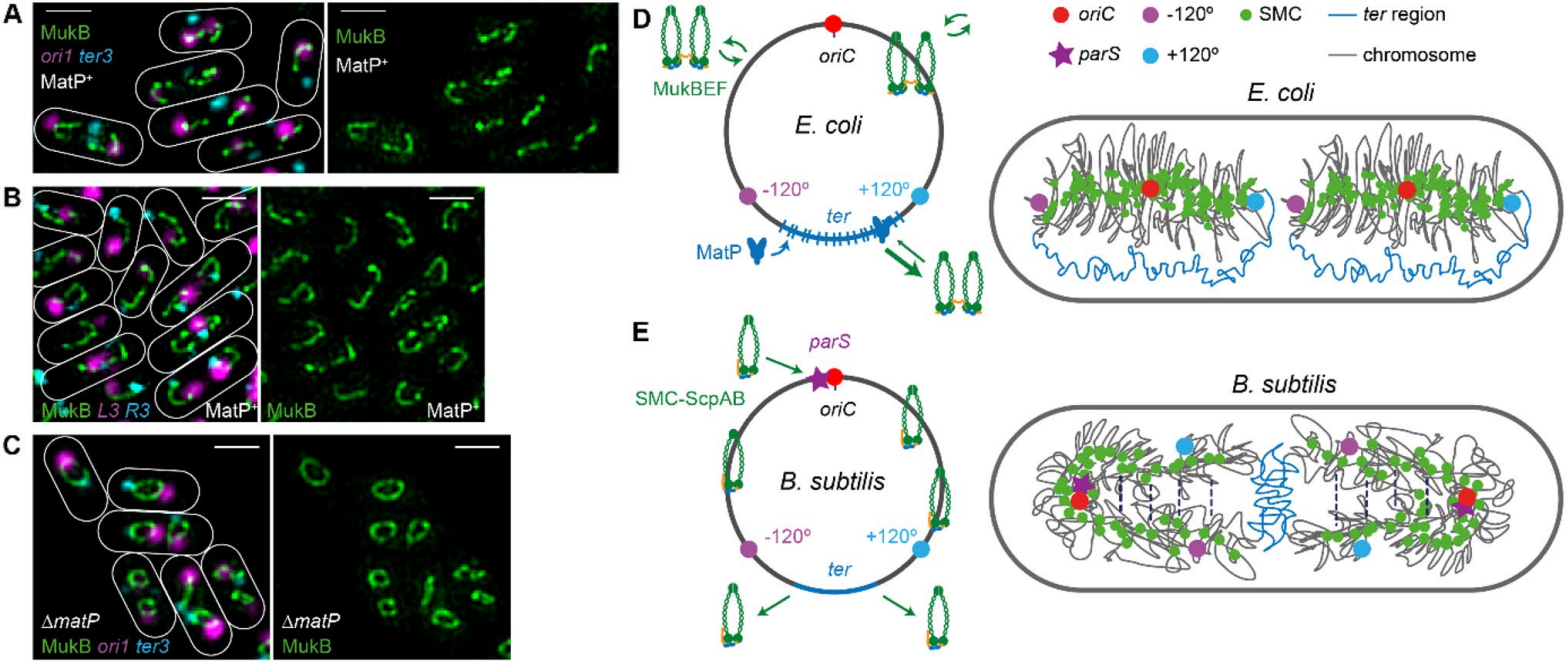
Bacterial chromosome organization*.* (**a–c**) Representative SIM microscopy images of *E. coli* cells showing MukBEF axial cores in relationship to the indicated genetic markers. Scale bars, 1 μm. **a** MatP^+^ cells with *ori1* and *ter3* markers. **b** MatP^+^ cells with *L3* and *R3* markers. **c** Δ*matP* cells with *ori1* and *ter3* markers. For further details, see (Mäkelä and Sherratt 2020). **d** *E. coli* chromosome showing 800 kbp ter region with *matS* sites (blue bars) that are bound by MatP (left) and depicted chromosome organization by MukBEF inside a cell with two chromosomes (right). **e** *B. subtilis* chromosome showing *parS* sites near *oriC* that with the help of ParAB recruit SMC-ScpAB to the chromosome (left) and depicted chromosome organization by SMC-ScpAB inside a cell with two chromosomes (right). Note that the experimental data suggest that the SMC complexes associated with two chromosome arms (blue and magenta dots) have their action coordinated (dashed lines, see text)

By modestly increasing *E. coli* chromosome occupancy of MukBEF, Mäkelä and Sherratt observed that fluorescent, functional MukBEF complexes formed a near continuous chromosomal axial core from which DNA loops of 20–50 kbp emanate (Mäkelä and Sherratt 2020) (Fig. 2a–c). The authors argue that in cells with wild-type MukBEF chromosome occupancy, a similar but more granular axial core also organizes the chromosome, consistent with the MukBEF complexes having identical residence times and diffusional properties in wild type and increased MukBEF occupancy cells. It was proposed that the formation of chromosome axial cores is a consequence of MukBEF loop extrusion, with the left and right chromosome arms (replichores) being individualized (Fig. 2a, b). The linear order of the chromosome was maintained relative to the axial cores, while the length of the axial core was > 1000 times shorter than the chromosomal DNA. MatP, which binds to 23 short *matS* sites in the 800 kbp replication termination region (*ter*) plays an essential role in determining the axial core shape. Deletion of *matP* led to formation of uniform circular axial cores, because MatP displaces MukBEF from *ter*, while MatP^+^ cells have linear cores as a consequence of MatP-directed displacement of MukBEF complexes from *ter* (Fig. 2c). Modeling using established parameters of MukBEF biology, and assuming a symmetric loop extrusion mechanism, provided an explanation of chromosome organization by axial cores (Fig. 2d). The models, which also assume that MukBEF loads randomly on all chromosomal regions, explain how MukBEF clusters colocalize with the replication origin region (*oriC*) in wild-type cells, while MukBEF clusters localize equally with all genetic regions tested in MatP^−^ cells.

Is the MukBEF-organized *E. coli* chromosome placed randomly within a cell? Early imaging studies showed that in new-born *E. coli* cells that have not initiated replication, the left and right replichores are organized into separate cell halves, while *oriC* is at midcell (Wang et al. 2006). After duplication, the two *oriC*s move to quarter positions and the chromosome arms remain in a translationally symmetric (Fig. 2d; left–right-left–right) configuration, which is inherited over generations. Facilitating this organization, *ter* is flexible and less compacted than rest of the chromosome, with different *ter* markers able to localize to distant regions of the same cell (Wang et al. 2005). MukBEF displacement from *ter* is essential for directing the chromosome arms to different cell halves*;* as in *matP* deletion strains, the distance between genetic markers in chromosome arms is reduced. Previous reports, which proposed that MatP compacts *ter* are inconsistent with the observations of Mäkelä and Sherratt and Hi-C chromosome conformation capture experiments that inform DNA-DNA contact lengths in ensemble analyses (Lioy et al. 2018). Reduced mobility or distances between some *ter* markers as a consequence of MatP action may instead be attributed to the partial anchoring of the chromosome to the divisome by a MatP-ZapB interaction (Espéli et al. 2012). This may also be reflected in the observation that linear MukBEF axial cores exhibit two different configurations: a ‘left-*oriC*-right’ configuration and one where both replichores point towards cell center, just prior to division (Fig. 2a, b). This anchoring may also influence the post-replication *ter* cohesion time, since *matP* deletion leads to earlier separation of *ter* markers (Nolivos et al. 2016), although it has been proposed that this results from increased local concentrations of the decatenase topoisomerase IV at *ter* in MatP-cells as a consequence of the specific interaction between MukB and topoisomerase IV (Hayama and Marians 2010; Li et al. 2010; Nolivos et al. 2016).

The action of MukBEF-MatP in individualization of chromosome arms, by directing left and right arms to opposite cell halves, contrasts with the situation in many bacteria that encode SMC–ScpAB complexes rather than MukBEF (e.g., *Bacillus subtilis* and *Caulobacter crescentus*). Indeed, MukBEF complexes and MatP-*matS* are largely confined to γ-proteobacteria (Brézellec et al. 2006). SMC-ScpAB action appears to ‘zip up’ the two chromosome arms promoting co-linearity of the two chromosome arms along the cell long axis, with *oriC*s located at the old pole in new borne cells and *ter* at the new pole (Fig. 2e). Intriguingly, Muk^−^ *E.coli* exhibit a similar organization (Danilova et al. 2007). This organization cannot be entirely attributed to SMC complex function, as it almost invariably occurs alongside ParAB*S* systems that are the main driving force behind chromosome segregation in many bacteria and which additionally recruit SMC complexes to the chromosome at specific *parS* sites near *oriC* (Fig. 1). Intriguingly, the γ-proteobacterium *Vibrio cholerae* encodes for two types of ParAB*S* system, each directed to a specific one of the two separate chromosomes, despite encoding MukBEF and MatP-*matS* (David et al. 2014; Demarre et al. 2014). Despite the different outcomes, the SMC action at the molecular level is likely to be similar in generating DNA loops dependent on ATP hydrolysis. The putative DNA loops could in principle form within a chromosome arm, with higher order interactions between SMC complexes on different arms aligning the two chromosome arms (Fig. 2d), consistent with the observation that halting SMC action on one arm impairs SMC progression on the other (Wang et al. 2017). Although MukBEF and indeed other SMC complexes play a pivotal role in establishing chromosome organization, other nucleoid associated proteins along with DNA supercoiling and molecular crowding also contribute to maintaining overall compact nucleoid organization.

## Future prospects

The mechanistic and functional differences between MukBEF and SMC–ScpAB complexes remain elusive, but in our opinion, it is likely that they both act through ATP hydrolysis-driven loop extrusion. The requirement of MukBEF dimers of dimers for function (Badrinarayanan et al. 2012; Rajasekar et al. 2019) provides a conceptually straightforward way of having a symmetrical loop extrusion mechanism, which in our model is essential for efficient lengthwise compaction (Mäkelä and Sherratt 2020). Whether other SMC complexes form dimers of dimers, or other-higher order cooperative structures, is hotly debated, although the apparent coordination of putative loop extrusion on the two *B. subtilis* arms could be explained by higher order SMC action. The first in vitro single-molecule studies of loop extrusion by condensin showed asymmetric loop extrusion (Ganji et al. 2018), a process not expected to be efficient in mitotic chromosome formation, although subsequent work hinted at how higher order activity by condensin can lead to overall symmetrical loop extrusion (Kim et al. 2020). A similar single-molecule study of loop extrusion by mammalian cohesin demonstrated symmetrical events (Davidson et al. 2019).

SMC complexes also have co-evolved with other chromosome binding proteins that can cooperate their activity with prospective loop formation; for example, MukBEF and MatP-*matS*, along with other proteins (Brézellec et al. 2006). Furthermore, it is likely that the interaction of the MukB hinge with the decatenase topoisomerase IV, likely has functional significance for coordinating the action of MukBEF with decatenation of newly replicated sister chromosomes. This interaction may be MukB specific, although there are reports of the functional interaction of eukaryote condensin with the decatenase TopoII (Coelho et al. 2003; Uhlmann 2016). Intriguingly, some bacteria have more than one type of SMC complex present in the same cells; for example, Pseudomonas species encode both SMC-ScpAB and the MukBEF homolog, MksBEF, with MksBEF genes being scattered among many Gm^−^ and Gm^+^ bacteria (Petrushenko et al. 2011). Nevertheless, the respective functions and cooperation between these orthologs remain unclear, especially as bacteria do not have clearly defined cell cycle stages or compartmentalisation, unlike eukaryotes, that would facilitate their regulated independent action on the chromosome. For example, in eukaryotes, cohesin is involved in chromosome organization in G1, but after replication establishment of cohesion between newly replicated sisters requires post-translational modification of cohesion, leading to a change in its properties. Similarly, mammalian mitotic chromosome compaction uses two condensins that are differentially expressed and compartmentalised (Yatskevich et al. 2019).

As in most biological systems, investigation of how SMC complexes function has been limited by the available assays. Early studies primarily exploited classical genetics and biochemistry, while later on new imaging techniques, particularly FISH-painting techniques, along with ensemble techniques like ChIP-seq and chromosome conformation capture techniques began to play important roles; in the latter case these can now be applied to single-cells [reviewed in (McCord et al. 2020)]. The repertoire of available techniques still limits functional advances, as do attempts to reconcile interpretations from these different techniques, all of which have limitations. Nevertheless, the development and exploitation of single-molecule techniques in vitro that appear to recapitulate the complete SMC complex reaction in which SMC complexes must undergo multiple conformational changes, with DNA being associated with at least two regions of the SMC complex, provide clues on the range of possible mechanistic actions that underpin chromosome conformation. They also provide scope to in vivo imaging techniques that can track the action of individual SMC complexes in live cells, and to cryoEM, alongside super-resolution techniques, that can reveal unprecedented level of details of the structure. For the future, the full range of techniques, alongside insightful questioning, will be required to unravel the complex relationship between SMC action and the emergent chromosome organization and dynamics.

## References

[CR1] Badrinarayanan A, Reyes-Lamothe R, Uphoff S (2012). In Vivo architecture and action of bacterial structural maintenance of chromosome proteins. Science.

[CR2] Brézellec P, Hoebeke M, Hiet MS (2006). DomainSieve: a protein domain-based screen that led to the identification of dam-associated genes with potential link to DNA maintenance. Bioinformatics.

[CR3] Coelho PA, Queiroz-Machado J, Sunkel CE (2003). Condensin-dependent localisation of topoisomerase II to an axial chromosomal structure is required for sister chromatid resolution during mitosis. J Cell Sci.

[CR4] Danilova O, Reyes-Lamothe R, Pinskaya M (2007). MukB colocalizes with the oriC region and is required for organization of the two *Escherichia coli* chromosome arms into separate cell halves. Mol Microbiol.

[CR5] David A, Demarre G, Muresan L (2014). The two *Cis*-acting sites, parS1 and oriC1, contribute to the longitudinal organisation of *Vibrio cholerae* chromosome I. PLoS Genet.

[CR6] Davidson IF, Bauer B, Goetz D (2019). DNA loop extrusion by human cohesin. Science.

[CR7] Demarre G, Galli E, Muresan L (2014). Differential management of the replication terminus regions of the two *Vibrio cholerae* chromosomes during cell division. PLoS Genet.

[CR8] Espéli O, Borne R, Dupaigne P (2012). A MatP-divisome interaction coordinates chromosome segregation with cell division in *E. coli*. EMBO J.

[CR9] Ganji AM, Shaltiel IA, Bisht S (2018). Real-time imaging of DNA loop extrusion by condensin. Science.

[CR10] Hayama R, Marians KJ (2010). Physical and functional interaction between the condensin MukB and the decatenase topoisomerase IV in *Escherichia coli*. Proc Natl Acad Sci.

[CR11] Hiraga S, Niki H, Ogura T (1989). Chromosome partitioning in *Escherichia coli*: novel mutants producing anucleate cells. J Bacteriol.

[CR12] Kakui Y, Uhlmann F (2018). SMC complexes orchestrate the mitotic chromatin interaction landscape. Curr Genet.

[CR13] Kim Y, Kim Y, Shi Z (2019). Human cohesin compacts DNA by loop extrusion. Science.

[CR14] Kim E, Kerssemakers J, Shaltiel IA (2020). DNA-loop extruding condensin complexes can traverse one another. Nature.

[CR15] Li Y, Stewart NK, Berger AJ (2010). *Escherichia coli* condensin MukB stimulates topoisomerase IV activity by a direct physical interaction. Proc Natl Acad Sci.

[CR16] Lioy VS, Cournac A, Marbouty M (2018). Multiscale structuring of the *E. coli* chromosome by nucleoid-associated and condensin proteins. Cell.

[CR17] Mäkelä J, Sherratt DJ (2020). Organization of the *Escherichia coli* chromosome by a MukBEF axial core. Mol Cell.

[CR18] McCord RP, Kaplan N, Giorgetti L (2020). Chromosome conformation capture and beyond: toward an integrative view of chromosome structure and function. Mol Cell.

[CR19] Nasmyth K (2001). Disseminating the genome: joining, resolving, and separating sister chromatids during mitosis and meiosis. Annu Rev Genet.

[CR20] Nolivos S, Upton AL, Badrinarayanan A (2016). MatP regulates the coordinated action of topoisomerase IV and MukBEF in chromosome segregation. Nat Commun.

[CR21] Palecek JJ, Gruber S (2015). Kite proteins: a superfamily of SMC/Kleisin partners conserved across bacteria, archaea, and eukaryotes. Structure.

[CR22] Paul MR, Hochwagen A, Ercan S (2019). Condensin action and compaction. Curr Genet.

[CR23] Petrushenko ZM, She W, Rybenkov VV (2011). A new family of bacterial condensins. Mol Microbiol.

[CR24] Rajasekar KV, Tang M, Baker R (2019). Dynamic architecture of the *Escherichia coli* structural maintenance of chromosomes (SMC) complex. MukBEF. Nucleic Acids Res.

[CR25] Uhlmann F (2016). SMC complexes: from DNA to chromosomes. Nat Rev Mol Cell Biol.

[CR26] Wang X, Possoz C, Sherratt DJ (2005). Dancing around the divisome: asymmetric chromosome segregation in *Escherichia coli*. Genes Dev.

[CR27] Wang X, Liu X, Possoz C, Sherratt DJ (2006). The two *Escherichia coli* chromosome arms locate to separate cell halves. Genes Dev.

[CR28] Wang X, Brandão HB, Le TBK (2017). Bacillus subtilis SMC complexes juxtapose chromosome arms as they travel from origin to terminus. Science.

[CR29] Wani S, Maharshi N, Kothiwal D (2018). Interaction of the *Saccharomyces cerevisiae* RING-domain protein Nse1 with Nse3 and the Smc5/6 complex is required for chromosome replication and stability. Curr Genet.

[CR30] Yatskevich S, Rhodes J, Nasmyth K (2019). Organization of chromosomal DNA by SMC complexes. Annu Rev Genet.

